# Quantitative assessment of aortic wall thickening disease by dual energy computed tomography angiography

**DOI:** 10.1186/s12880-026-02605-8

**Published:** 2026-07-24

**Authors:** Na Li, Jia Liu, Qinyue Luo, Lijie Zhang, Kailu Zhang, Yu Feng, Heshui Shi

**Affiliations:** 1 Department of Radiology, Union Hospital, Tongji Medical College, Huazhong University of Science and Technology, 1277 Jiefang Rd., Wuhan, 430022 China; 2 Hubei Provincial Clinical Research Center for Precision Radiology & Interventional Medicine, Wuhan, 430022 China; 3 Hubei Province Key Laboratory of Molecular Imaging, Wuhan, 430022 China; 4 Department of Radiology, Jingshan People’s Hospital, Jingshan, 431800 China

**Keywords:** Dual energy, Thrombus, Hematoma, Atherosclerosis, Computed tomography, Aorta

## Abstract

**Objectives:**

A broad spectrum of etiologies can lead to aortic wall thickening. This study aimed to evaluate the utility of quantitative parameters derived from dual-layer computed tomography (CT) in differentiating intramural hematoma (IMH), non-calcified plaques, and aortic thrombosis, and to explore their associations with laboratory inflammatory markers.

**Methods:**

365 patients were retrospectively enrolled, including atherosclerosis (*n* = 98), aortic dissection (*n* = 126), and IMH (*n* = 141). Laboratory indicators were collected from the electronic medical record system. Regions of interest (ROI) of non-calcified plaques, thrombi, hematomas, and periaortic adipose tissue around lesions were outlined. The lesion enhancement values and degree of enhancement were calculated from the CT values in polyenergetic and virtual non-contrast image. The slope of the energy spectrum curve was determined using the formula: K = (CT_40keV_ - CT_100keV_) / 60. Normalization was performed for the effective atomic number (Z), iodine density (ID), and iodine non-water density (IW) using the aortic lumen as the standard reference, resulting in normalized Z values, normalized iodine density (NID), and normalized iodine non-water density (NIW).

**Results:**

The enhancement values and degrees of enhancement differed among the three lesions (all *P* < 0.05). Differences in Z value, ID, IW, and K were observed among non-calcified plaques, thrombi, and hematomas (all *P* < 0.05). The NID of hematoma was the largest, while the NIW of thrombus was the smallest. Normalized Z value of non-calcified plaque was significantly smaller than those of thrombus and hematoma (both *P* < 0.05), with no difference between thrombus and hematoma (*P* > 0.05). The Z_periaortic fat_ pre- and post-normalization were significantly smaller around the plaques than around the thrombi and hematomas (both *P* < 0.05). Significant correlations were found between aortic wall normalized Z_lesion_, Z_periaortic fat_, and laboratory parameters, including neutrophil % and neutrophil count, lymphocyte % and lymphocyte count, C-reactive protein, and D-dimer.

**Conclusions:**

Quantitative spectral CT parameters show promise for the non-invasive identification of aortic wall lesions. The weak correlations with laboratory inflammatory markers suggest a potential but limited link between imaging parameters and local inflammation. These exploratory findings require further validation. If confirmed in future studies, this approach may complement conventional CTA and guide targeted therapy.

**Clinical trial number:**

Not applicable.

**Supplementary Information:**

The online version contains supplementary material available at https://doi.org/10.1186/s12880-026-02605-8.

## Introduction

The aorta is the largest artery in the human body. Aortic diseases, including aortic atherosclerosis (AS), acute aortic syndrome (AAS), mural thrombus, and inflammatory lesions of the aorta, can cause thickening of the aortic wall. Primary aortic mural thrombosis is challenging to diagnose and is associated with high mortality and a substantial risk of complications. Consequently, early detection and timely intervention are critical to achieving a favorable prognosis [1]. Diagnostic imaging is crucial for identifying aortic lesions and determining appropriate interventions.

Computed tomography angiography (CTA) is the preferred imaging modality for aortic diseases [2]. However, in some cases, conventional computed tomography (CT) may be difficult to distinguish intramural hematoma (IMH) from wall thickening caused by AS, mural thrombus, or aortic dissection (AD) with complete thrombosis of the false lumen [3, 4].

Dual-layer detector spectral CT (DLCT) is capable of acquiring material attenuation information at two different energies to identify and classify different tissues based on the difference in energy absorption [5]. Virtual monoenergetic images (VMIs) from dual-energy CT (DECT) enhance soft tissue and iodine contrast, improving arterial visualization and characterization [6, 7]. Studies have shown that high-energy VMI reduces image noise, which helps minimize artifacts and improves the characterization of vascular plaques [8]. Conversely, low-energy VMI can increase inter-tissue contrast, thereby enhancing iodine contrast. For patients with lower extremity deep vein thrombosis, 40 keV VMI offers better image quality and diagnostic accuracy than conventional mixed-energy CT [9]. Previous studies have shown that DECT can assess the composition of atherosclerotic plaques in carotid [10] and coronary arteries [11, 12], but there are fewer studies on the differential diagnosis of aortic diseases. In addition, laboratory tests are convenient, rapid and important in the differential diagnosis of the aortic disease, but the indicators are not specific.

Thus, this study aims to investigate the value of quantitative parameters from DLCT in the differential diagnosis of IMH, non-calcified plaques and thrombi in the aorta, and the relationship between the characteristic parameters and laboratory inflammatory markers.

## Patients and methods

### Study population

This retrospective study enrolled patients with suspected AAS who underwent whole aortic CTA on dual-layer detector spectral CT at Wuhan Union Hospital between June 2020 and September 2023. The study was approved by the hospital’s ethics committee and patients were waived from informed consent.

The inclusion criteria were as follows: unanimous diagnosis of AD with partial thrombosis of the false lumen, IMH of the aorta, and aortic AS, confirmed by two physicians with at least 5 years of experience in cardiovascular imaging diagnosis. The overall exclusion criteria were as follows: (1) aortic surgery; (2) traumatic aortic injury, congenital disease of the aorta, and inflammatory lesions; (3) normal aorta; (4) combined acute coronary syndrome or oncologic disease; (5) basic clinical information is missing; (6) CT images could not be analyzed. Meanwhile, each group was also required to individually fulfill the following exclusion criteria: the thrombus group excluded AD without false-lumen thrombus or with complete thrombosis; the hematoma group excluded concurrent AD; and the plaque group excluded concurrent AD or IMH.

Finally, 365 patients were enrolled in this study, including 126 patients with AD (thrombus group), 141 patients with IMH (hematoma group), and 98 patients with AS (plaque group).

### Clinical data collection

General clinical data and laboratory indices of the patients including sex, age, comorbidities (hypertension, diabetes mellitus, dyslipidemia), history of smoking, history of alcohol consumption, fasting blood glucose, total cholesterol, triglycerides, white blood cell count, neutrophil %, lymphocyte %, neutrophil count, lymphocyte count, C-reactive protein (CRP), and D-dimer. were collected from the electronic medical record system of our hospital.

Hypertension was defined as systolic blood pressure ≥ 140 mmHg, diastolic blood pressure ≥ 90 mmHg, or use of antihypertensive medication. Diabetes mellitus was defined as fasting blood glucose ≥ 7.0 mmol/L or use of hypoglycemic drugs. Dyslipidemia was defined as one of the following: total cholesterol ≥ 5.7 mmol/L, low density lipoprotein (LDL) cholesterol ≥ 4.1 mmol/L, triglycerides ≥ 1.7 mmol/L, or use of cholesterol-lowering drugs.

### CT imaging acquisition

Aortic CTA was performed on dual-layer spectral detector CT (IQon, Philips Healthcare, Best, The Netherlands). The scanning range was from the level of thoracic inlet to pubic symphysis. Electrocardiographic gating was applied, with the region of interest (ROI) placed at the level of the thoracoabdominal junction. The scan was automatically triggered when the threshold within the ROI reached 130 HU.

The scanning parameters were as follows: tube voltage of 120 kVp, tube current determined by automatic tube current modulation (DoseRight 3D-DOM, Philips Healthcare, Best, The Netherlands), detector collimation width of 64 × 0.625 mm, pitch of 0.969, rotation time of 0.27 s, slice thickness of 1 mm, and increment of 0.75 mm. All patients were injected with iodixanol (320 mg I/mL, Bayer, Shanghai, China) via the median cubital vein. Contrast injection was performed with an injection rate of 2.3–3.7 ml/s and a contrast dosage of 36–54 ml. The spectral based images (SBI) were uploaded to a Philips post-processing workstation.

### Image reconstruction and post-processing

The SBI data was loaded on a Philips post-processing workstation (Philips Intellispace portal), and the projection spatial spectral reconstruction technique was utilized to obtain virtual non-contrast image (VNC), 40 keV virtual monoenergetic image (VMI), 100 keV VMI, and 120 kVp polyenergetic image (PI) using hybrid iterative reconstruction. The entire aorta was scanned, avoiding beam-hardening artifacts, and the thickest aortic wall in cross section with good image quality was selected to outline the ROI of the lesion.

Plaque ROIs were outlined taking care, to avoid calcified plaques and to select the non-calcified portion of mixed plaques. Thrombus and hematoma ROIs were outlined, taking care to place the ROI in the center of the lesion. Simultaneously, ROIs were placed in the lumen (true lumen in patients with AD) and in the periaortic adipose tissue surrounding the lesion at the same level, as described below: ROIs were sketched on VMI_40keV_ and used in VNC, PI, VMI_100keV_, effective atomic number image, iodine density and iodine non-water density images by copy and paste to ensure that ROIs at the same site were consistent in size, morphology and location (Supplementary Fig. 1). The area of the ROIs was > 4 mm^2^. The average CT value, standard deviation (SD), and spectral parameters were recorded, including: effective atomic number (Z), iodine density (ID) and iodine non-water density (IW). Two measurements were obtained at each site and then averaged. The slope of the energy spectrum curve was calculated according to the formula:

$$K\,=\,\frac{CT_{40\mathrm{keV}}\,-\,CT_{100\mathrm{keV}}}{60}$$

Perivascular adipose tissue (PVAT) was defined as adipose tissue located within a 5 mm radial distance from the outer aortic wall, with CT attenuation values ranging from -190 to 30 HU [13]. To remove the effects of contrast injection and patient, the Z-value, ID, IW of the wall lesion were normalized by the aortic lumen (true lumen) to obtain a normalized Z-value, normalized iodine density (NID), and normalized iodine non-water density (NIW). The ΔCT_VNC_ was calculated as the difference in CT values between the lesion and the lumen, and the enhancement value (Δ CT_lesion_) and degree of enhancement (Δ CT_lesion_ %) of aortic wall lesions were determined using the CT values of lesions in PI and VNC. The following formulas were as follows:

$$\Delta\mathrm{CT\,}_{\mathrm{VNC\,}}\,=\,\mathrm{VNC\,}_{\mathrm{lumen}}\,-\,\mathrm{VNC\,}_{\mathrm{lesion}}$$

$$\Delta\mathrm{CT\,}_{\mathrm{lesion}}\,=\,\mathrm{PI\,}_{\mathrm{lesion}}\,-\,\mathrm{VNC\,}_{\mathrm{lesion}}$$

$$\Delta\mathrm{CT\,}_{\mathrm{lesion}}\%\,=\,\Delta\mathrm{CT\,}_{\mathrm{lesion}}\,/\,\mathrm{VNC\,}_{\mathrm{lesion}}$$

### Repeatability

Thirty subjects (10 from each group) were randomly selected from the overall study cohort. The values were measured independently by two radiologists with more than 5 years of experience in the diagnosis of cardiovascular diseases. Two independent observers (Observer 1 and Observer 2) assessed aortic wall lesions and performed ROI delineation and measurements according to the methods described above. Observer 1 re-evaluated the aorta and performed a second measurement after an interval of > 2 weeks. To ensure concordance of the target lesion, both observers referenced the anatomical location initially identified by Observer 1; however, the final measurement slice was selected independently by each observer. Inter- and intra-observer intraclass correlation coefficients (ICCs) of the values on VMI_40keV_, VNC, PI, VMI_100keV_, effective atomic number image, iodine density, and iodine non-water density images were calculated separately to assess repeatability.

### Statistical analysis

SPSS 26.0 was used for all statistical analysis. The normality of all continuous variables was assessed using the Shapiro–Wilk test. Normally distributed data were presented as mean ± standard deviation (SD), while non-normally distributed data were expressed as median with interquartile range (IQR). Categorical variables were summarized as frequencies and percentages. Comparisons among the three groups were performed using one-way analysis of variance (ANOVA) for normally distributed data and the Kruskal–Wallis H test for non-normally distributed data. When the test indicated a significant difference, pairwise comparisons were conducted using the Bonferroni–Dunn post-hoc correction for multiple comparisons. Chi-squared test was used to compare between multiple groups of count data. The receiver operating characteristic curve (ROC) was used to assess the efficacy of the quantitative parameters of spectral CT for disease identification. Comparison between ROC curves was performed using the Delong test. The Pearson correlation coefficient was applied when both variables were approximately normally distributed; otherwise, Spearman’s rank correlation coefficient was used. The difference was statistically significant at *P* < 0.05.

## Results

### Demographic information

A total of 365 patients were included in this study, with a mean age of 58.29 ± 11.9 years. 308 patients (84.4%) were male (Table 1). The mean systolic and diastolic blood pressures on admission were 138.86 ± 20.2 mmHg and 82.93 ± 12.25 mmHg, respectively, of which 172 (47.1%) had hypertension. 126 (34.5%) patients had a history of smoking and 84 (23%) had a history of alcohol consumption. Mean fasting blood glucose at admission was 6.15 ± 2.18 mmol/L; of these, 95 (26%) were combined with diabetes mellitus. 167 (45.8%) had dyslipidemia. The three groups were statistically different in age, height, weight, hypertension, diabetes mellitus, dyslipidemia, and history of alcohol consumption (all *P* < 0.05). In addition to fasting blood glucose and triglyceride levels, the total cholesterol, white blood cell count, neutrophil percentage, lymphocyte percentage, neutrophil count, lymphocyte count, CRP, and D-dimer showed statistically significant differences among the three groups (all *P* < 0.05).

**Table 1:** General clinical data and laboratory indicators of the patient

indexes	Total (*n* = 365)	plaque group (*n* = 98)	thrombus group (*n* = 126)	hematoma group (*n* = 141)	*P* values
Age (years)	58.29 ± 11.9	63.96 ± 8.75	54.34 ± 13.57	57.88 ± 10.67	**< 0.001**
Male (n, %)	308 (84.4%)	81 (82.7%)	110 (87.3%)	117 (83%)	0.536
Height (cm)	168.77 ± 6.72	167.21 ± 7.07	169.94 ± 6.29	169.03 ± 6.62	**0.018**
Weight (kg)	70.14 ± 12.08	67.16 ± 11.2	71.25 ± 12.99	71.54 ± 11.66	**0.040**
BMI (kg/m^2^)	24.54 ± 3.5	23.93 ± 3.15	24.59 ± 3.66	24.97 ± 3.58	0.098
Hypertension (n, %)	172 (47.1%)	51 (52.0%)	42 (33.3%)	79 (56%)	**0.001**
Systolic blood pressure (mmHg)	138.86 ± 20.2	132.03 ± 22.81	141.82 ± 18.57	141.73 ± 18.11	**0.001**
Diastolic blood pressure (mmHg)	82.93 ± 12.25	79.96 ± 12.08	84.56 ± 13.37	83.91 ± 11.08	**0.020**
Smoker (n, %)	126 (34.5%)	37 (37.8%)	35 (27.8%)	54 (38.3%)	0.144
Drinker (n, %)	84 (23%)	15 (15.3%)	26 (20.6%)	43 (30.5%)	**0.017**
Diabetes mellitus (n, %)	95 (26.0%)	34 (34.7%)	24 (19%)	37 (26.2%)	**0.03**
FBG (mmol/L)	5.1 (4.7 ~ 6)	5.05 (4.7 ~ 5.5)	4.9 (4.4 ~ 7.43)	6.05 (5.13 ~ 6.38)	0.445
Dyslipidemia (n, %)	167 (45.8%)	65 (66.3%)	51 (40.5%)	51 (36.2%)	**< 0.001**
Total cholesterol (mmol/L)	4.18 ± 1.11	3.9 ± 0.79	4.38 ± 1.03	4.29 ± 1.42	**0.035**
Triglyceride (mmol/L)	1.17 (1 ~ 1.59)	1.20 (0.94 ~ 1.53)	1.43 (0.99 ~ 1.95)	1.08 (1.02 ~ 1.15)	0.353
White blood cell count (10^9/L)	8.02 ± 3.14	6.74 ± 2.02	8.06 ± 3.94	8.96 ± 2.75	**< 0.001**
Neutrophil % (%)	64.27 (61.6 ~ 77.6)	64.44 (63.43 ~ 75.45)	63.51 (56.7 ~ 78.2)	68.54 (60.54 ~ 84.00)	**< 0.001**
Lymphocyte % (%)	18.97 ± 9.72	25.46 ± 8.7	18.1 ± 9.91	14.73 ± 7.51	**< 0.001**
Neutrophil count (10^9/L)	5.99 ± 4.11	4.31 ± 1.71	6.06 ± 3.8	7.22 ± 5.12	**< 0.001**
Lymphocyte count (10^9/L)	1.3 (1 ~ 1.7)	1.24 (1 ~ 1.39)	1.54 (1 ~ 1.7)	1.56 (0.88 ~ 1.84)	**< 0.001**
CRP (mg/L)	10.3 (3.13 ~ 38.44)	3.49 (3.13 ~ 16.38)	17.1 (3.3 ~ 80)	24.85 (17.49 ~ 35.08)	**< 0.001**
D-dimer (ng/mL)	0.68 (0.39 ~ 1.81)	0.39 (0.278 ~ 1.06)	1.21 (0.63 ~ 7.77)	2.42 (0.77 ~ 4.54)	**< 0.001**

Note: BMI: body mass index; FBG: fasting blood glucose; CRP: C-reaction protein

### Comparison of CT values among three groups

**Fig. 1:**
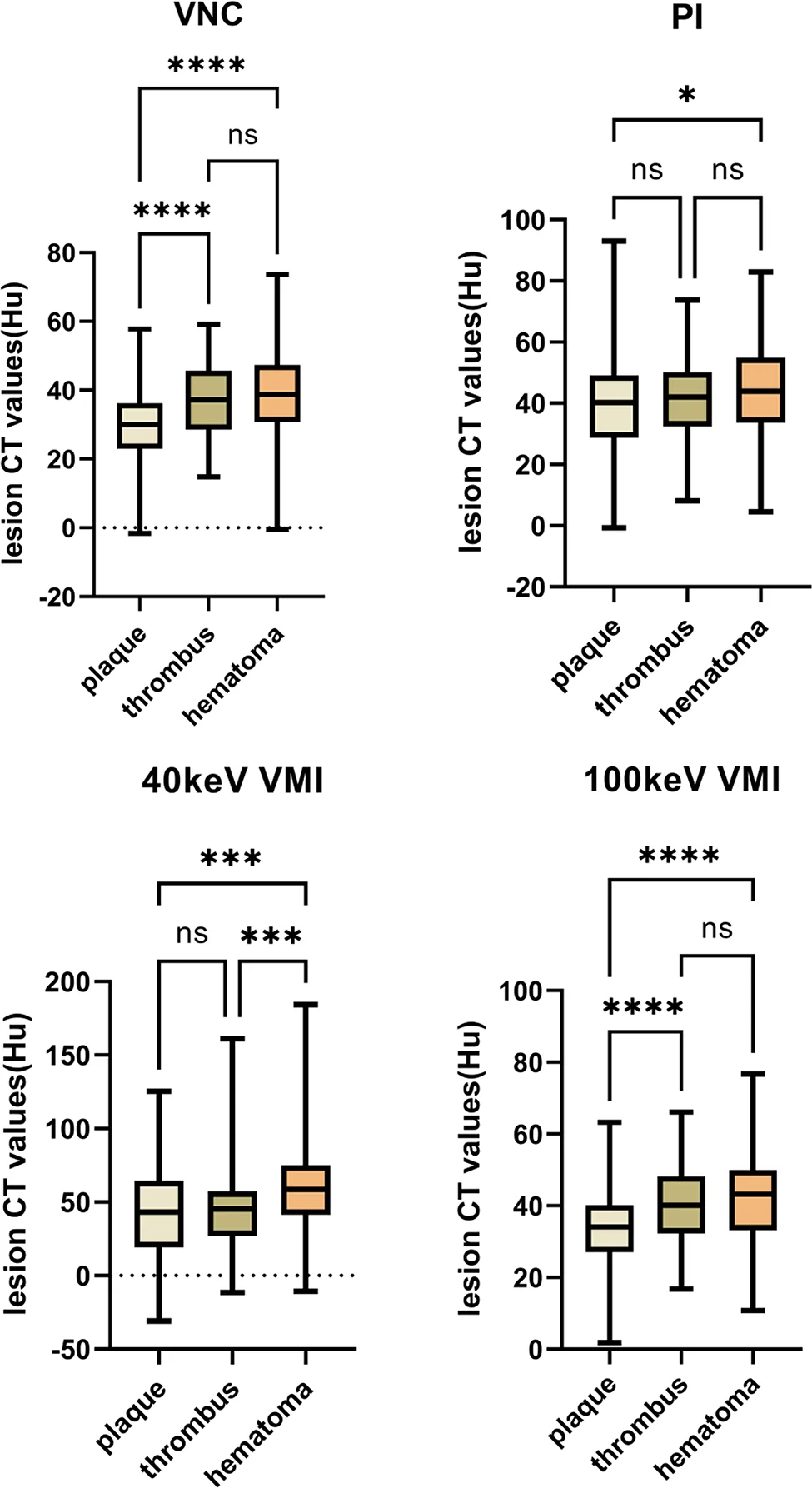
CT values of aortic wall lesions in different images. VNC: virtual non-contrast image; PI: polyenergetic image; VMI: virtual monoenergetic image. ^ns^*P* > 0.05; *P < 0.05; **P < 0.01; ***P < 0.001; **** *P* < 0.0001

**Fig. 2:**
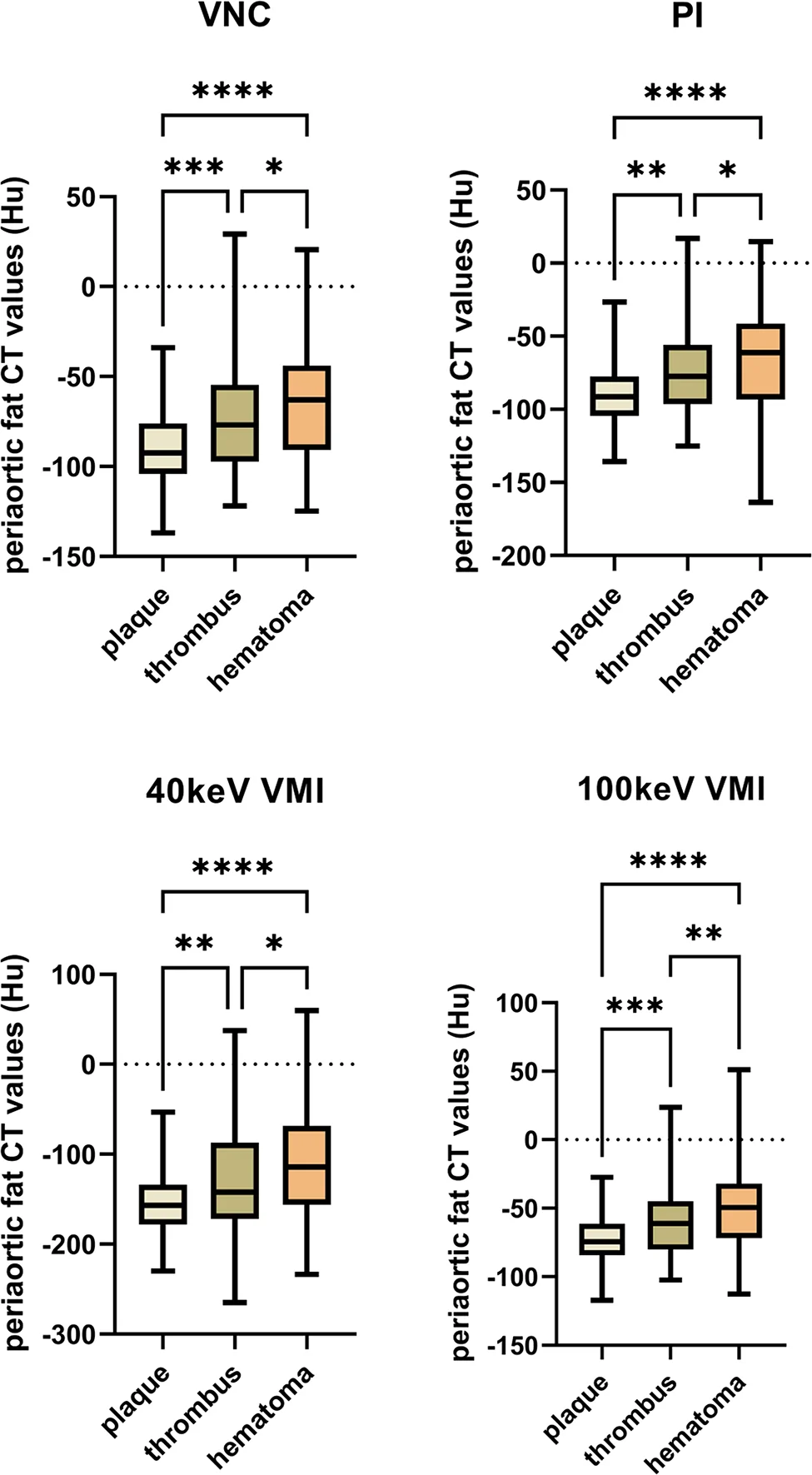
CT values of periaortic fat in different images. VNC: virtual non-contrast image; PI: polyenergetic image; VMI: virtual monoenergetic image. *P < 0.05; **P < 0.01; ***P < 0.001; ****P < 0.0001

In the four images (VNC, PI, VMI_40keV_ and VMI_100keV_), statistically significant differences were observed among the three groups in CT attenuation of aortic wall lesions, lumen, and periaortic fat (Fig. 1). The CT values of aortic wall lesions in all four images indicated that hematoma had a higher attenuation value than thrombus and non-calcified plaque. The difference in attenuation values among the three lesions was statistically significant (*P* < 0.05). For periaortic fat attenuation, a stepwise decrease was observed from the hematoma group to the thrombus group to the plaque group, and the difference between any two groups was statistically significant (*P* < 0.05) (Fig. 2).

**Table 2:** Contrast enhancement patterns of the three lesion groups

	plaque group (*n* = 98)	thrombus group (*n* = 126)	hematoma group (*n* = 141)	*P* values
△CT_lesion (Hu)_	6.7 (3.2 ~ 14.53)	2.55 (-0.4 ~ 7.75) ^a^	3.5 (-0.25 ~ 9.5) ^a^	**< 0.001**
△CT_lesion_% (%)	21.77 (11.21 ~ 49.05)	7.5 (-1.07 ~ 22.65) ^a^	9.25(-0.72 ~ 23.19) ^a^	**< 0.001**

Note: a: compared with the plaque group, *P* < 0.05

**Table 3:** Distribution of ∆CT_VNC_ and ∆CT _lesion_ for the three lesions

	△CT_VNC_ (Hu)			△CT_lesion_ (Hu)		
	> 0	= 0	< 0	> 0	= 0	< 0
plaque (*n* = 98)	87	0	11	87	0	11
thrombus (*n* = 126)	99	2	25	91	1	34
hematoma (*n* = 141)	99	0	42	104	0	37
Total (*n* = 365)	285	2	78	282	1	82

Note: Data expressed as frequency

The enhancement values and degree of enhancement differed among the three lesions of the aortic wall (both *P* < 0.05). Specifically, non-calcified plaques had significantly higher enhancement values than did thrombus and hematoma (both *P* < 0.05), while there was no significant difference between thrombus and hematoma (*P* > 0.05) (Table 2).

The distribution of ∆CT_VNC_ and ∆CT_lesion_ for the three lesions was shown in Table 3. In VNC, 285 patients had higher luminal attenuation than wall lesion attenuation. In 82 patients, the lesion attenuation values in VNC were greater than the attenuation values in PI.

### Comparison of spectral CT characteristic parameters among three groups

Differences in Z-value, ID, IW and slope K among non-calcified plaques, thrombi and hematomas were statistically significant (all *P* < 0.05) (Table 4). The Z-value, ID, IW and K of hematoma were greater than those of thrombus (all *P* < 0.05). Additionally, non-calcified plaque showed greater IW than did thrombi (*P* < 0.05), whereas no significant differences were observed between non-calcified plaques and thrombi in terms of Z value, ID and K (all *P* > 0.05). Differences were also found between the three normalized spectral CT characteristic parameters of aortic wall lesions and Z_periaortic fat_ (all *P* < 0.05). NID for hematoma was the largest and NIW for thrombus the smallest. Unlike before normalization, normalized Z-values for non-calcified plaque were significantly smaller than those of thrombus and hematoma (both *P* < 0.05), whereas there was no difference between hematoma and thrombus (*P* > 0.05). Both pre- and post-normalization Z_periaortic fat_ were significantly smaller in the plaque group than in the thrombus and hematoma groups (both *P* < 0.05).

**Table 4:** Comparison of CT characteristic parameters of wall lesions, lumen and periaortic fat in three groups

indexes	Total (*n* = 365)	plaque group (*n* = 98)	thrombus group (*n* = 126)	hematoma group (*n* = 141)	*P* values
Z_lesion_	7.33 (7.21 ~ 7.49)	7.33 (7.17 ~ 7.54)	7.28 (7.18 ~ 7.41)	7.37 (7.25 ~ 7.53) ^b^	**< 0.001**
ID_lesion_	0.11 (0 ~ 0.35)	0.11 (0 ~ 0.43)	0.06 (0 ~ 0.23)	0.17 (0.02 ~ 0.42) ^b^	**< 0.001**
IW_lesion_	0.14 (0 ~ 0.4)	0.17 (0 ~ 0.5)	0.08 (0 ~ 0.23) ^a^	0.22 (0.02 ~ 0.44) ^b^	**0.002**
Z_lumen_	10.86 (10.38 ~ 11.31)	11.35 (10.68 ~ 11.72)	10.67 (10.07 ~ 10.99) ^a^	10.74 (10.35 ~ 11.28) ^a^	**< 0.001**
ID_lumen_	9.4 ± 2.85	10.97 ± 2.95	8.48 ± 2.5 ^a^	9.12 ± 2.63 ^a^	**< 0.001**
IW_lumen_	9.37 ± 2.82	10.99 ± 2.95	8.21 ± 2.21^a^	9.13 ± 2.63 ^ab^	**< 0.001**
Z_periaortic fat_	6.36 ± 0.5	6.19 ± 0.41	6.38 ± 0.5 ^a^	6.48 ± 0.52 ^a^	**< 0.001**
ID_periaortic fat_	0 (0 ~ 0)	0 (0 ~ 0)	0 (0 ~ 0)	0 (0 ~ 0)	0.066
IW_periaortic fat_	-0.03 (-0.18 ~ 0.08)	-0.03 (-0.29 ~ 0.14)	-0.06 (-0.16 ~ 0.03)	-0.02 (-0.14 ~ 0.08)	0.179
K_lesion_	0.14 (-0.11 ~ 0.45)	0.14 (-0.17 ~ 0.53)	0.05 (-0.17 ~ 0.29)	0.22 (-0.02 ~ 0.53) ^b^	**0.001**
Normalized Z_lesion_	0.68 (0.65 ~ 0.71)	0.65 (0.62 ~ 0.68)	0.69 (0.66 ~ 0.73) ^a^	0.69 (0.65 ~ 0.72) ^a^	**< 0.001**
NID_lesion_	0.01 (0 ~ 0.04)	0.01 (0 ~ 0.04)	0.01 (0 ~ 0.03)	0.02 (0 ~ 0.04) ^b^	**0.002**
NIW_lesion_	0.02 (0 ~ 0.04)	0.02 (0 ~ 0.04)	0.01 (0 ~ 0.02) ^a^	0.02 (0 ~ 0.05) ^b^	**< 0.001**
Normalized Z_periaortic fat_	0.58 (0.54 ~ 0.63)	0.55 (0.52 ~ 0.58)	0.6 (0.55 ~ 0.64) ^a^	0.6 (0.56 ~ 0.65) ^a^	**< 0.001**

Note: a: compared with the plaque group, *P* < 0.05; b: compared with the thrombus group, *P* < 0.05

### Evaluation of the efficacy of quantitative parameters of spectral CT for the differential diagnosis of three lesions

The AUCs for differentiating non-calcified plaques using enhancement value and degree of enhancement were 0.668 and 0.688, respectively, with a sensitivity higher than 75% and a specificity lower than 60% (Table 5). The diagnostic efficacy of lesion CT values in VNC and VMI_100keV_ for plaques was comparable to the first two, with higher sensitivity and lower specificity. The diagnostic performance of normalized Z_lesion_ and normalized Z_periaortic fat_ for non-calcified plaques was slightly higher than the enhancement value and the degree of enhancement, with a specificity improvement of more than 10%. The AUCs for the diagnosis of thrombus were 0.619 and 0.598 for IW_lesion_ and NIW_lesion_, respectively, with a sensitivity of 74.8% and 70.1% and comparable specificity. Wall lesion CT values in VMI_40keV_ and periaortic fat CT values in VNC, PI, VMI_40keV_, and VMI_100keV_ showed moderate diagnostic efficacy for hematomas, with no statistically significant differences among these indices (all *P* > 0.05).

**Table 5:** Diagnostic performance of aortic wall lesions by quantitative parameters of spectral CT

Indexes	AUC	*P values*	Cut-off values	Sensitivity (%)	Specificity (%)	Delong test
Identification of no-calcified plaques						
VNC lesion CT values (Hu)	0.698	**< 0.001**	≤ 36.6	76.5	43.4	Z_periaortic fat_ vs.normalized Z_periaortic fat_: *P* < 0.01; *P* > 0.05 between the remaining two groups
VMI_100keV_ lesion CT values (Hu)	0.684	**< 0.001**	≤ 40.2	75.5	55.1	
△CT_lesion_ (Hu)	0.668	**< 0.001**	≥ 2.8	79.6	48.3	
△CT_lesion_% (%)	0.688	**< 0.001**	≥ 11.3	75.5	57.7	
Z_periaortic fat_	0.647	**< 0.001**	≤ 6.4	72.4	55.4	
Normalized Z_lesion_	0.7	**< 0.001**	≤ 0.67	66.3	69.7	
Normalized Z_periaortic fat_	0.736	**< 0.001**	≤ 0.56	68.4	70.8	
Identification of thrombus						
IW_lesion_	0.619	**< 0.001**	≤ 0.20	74.8	51	*P* = 0.016
NIW_lesion_	0.598	**0.003**	≤ 0.02	70.1	51	
Identification of hematomas						
VMI_40keV_ lesion CT values (Hu)	0.645	**< 0.001**	≥ 53.6	56.7	67.4	Both *P* > 0.05 for groups
VNC periaortic fat CT values (Hu)	0.661	**< 0.001**	≥ -70.9	61	68.3	
PI periaortic fat CT values (Hu)	0.651	**< 0.001**	≥ -68.3	58.2	70.1	
VMI_40keV_ periaortic fat CT values (Hu)	0.645	**< 0.001**	≥ -133.8	64.5	62.5	
VMI_100keV_ periaortic fat CT values (Hu)	0.668	**< 0.001**	≥ -56.7	59.6	70.5	

Note: VNC: virtual non-contrast image; PI: polyenergetic image; VMI: virtual monoenergetic image; IW: iodine non-water density; NIW: normalized iodine non-water density

### Correlation between characteristic parameters of spectral CT and laboratory indicators

The correlations between the spectral CT characteristic parameters of aortic wall lesions and laboratory indices were shown in Table 6. White blood cell counts showed a significant positive correlation with Z_periaortic fat_. Neutrophil %, neutrophil count, CRP, and D-dimer were all positively correlated with Z_periaortic fat_ and normalized Z_lesion_. Lymphocyte % and lymphocyte count were all significantly negatively correlated with Z_periaortic fat_ and normalized Z_lesion_. Although the observed correlations were weak, they were statistically significant, and the absolute correlation coefficients (|r|) ranged from 0.12 to 0.29.

**Table 6:** The relationship between spectral parameters and laboratory indicators

Laboratory indicators	Spectral parameters	*r* values	*P* values
White blood cell count	Z_periaortic fat_	0.123	**0.034**
Neutrophil %	Z_periaortic fat_	0.196	**0.001**
	Normalized Z_lesion_	0.229	**< 0.001**
Lymphocyte %	Z_periaortic fat_	-0.210	**< 0.001**
	Normalized Z_lesion_	-0.243	**< 0.001**
Neutrophil count	Z_periaortic fat_	0.14	**0.016**
	Normalized Z_lesion_	0.142	**0.015**
Lymphocyte count	Z_periaortic fat_	-0.187	**0.001**
	Normalized Z_lesion_	-0.231	**< 0.001**
CRP	Z_periaortic fat_	0.249	**0.004**
	Normalized Z_lesion_	0.290	**0.001**
D-dimer	Z_periaortic fat_	0.277	**< 0.001**
	Normalized Z_lesion_	0.258	**0.001**

Note: CRP: C-reaction protein

### Reproducibility

The ICCs in the intra-observer analysis were 0.919, 0.951, 0.978, 0.948, 0.958, 0.967, 0.945 for the CT values on VMI_40keV_, VNC, PI, VMI_100keV_, Z values, ID and IW, respectively. Finally, the ICCs were 0.892, 0.927, 0.980, 0.921, 0.975, 0.985, 0.946 for the CT values on VMI_40keV_, VNC, PI, VMI_100keV_, Z values, ID and IW, respectively, in the inter-observer analysis.

## Discussion

Aortic wall thickening can be caused by intramural hematomas, atherosclerotic plaques, and thrombosis. It is important to identify these diseases early and accurately to properly manage them. This is crucial for risk stratification, clinical intervention, prognostic assessment, and the prevention of complications. Spectral CT offers a new diagnostic approach with a broad range of objective, quantitative, and non-invasive parameters for assessing diseases [14, 15]. In this study, we found that the normalized Z_lesion_ can assist in the identification of non-calcified plaques from thrombi and hematomas; the slope of the energy spectrum curve and NID can assist in the identification of thrombi and hematomas, and the NIW can assist in the identification of thrombi from non-calcified plaques and hematomas.

The results showed that the CT values of hematomas were greater than those of non-calcified plaques in VNC, PI, VMI_40keV_ and VMI_100keV_. Hematomas consist of components such as hemoglobin and ferrous haemoflavin, which can cause an increase in x-ray attenuation [16]. Plaques result from endothelial lipid deposition and therefore differ more markedly from hematoma attenuation values. There were no difference between hematoma and thrombus attenuation values in VNC, PI and VMI_100keV_, and only in VMI_40keV_, the hematoma attenuation values was significantly greater than the thrombus. This may be due to the following two reasons. Firstly, low keV VMI enhances lesion saliency and increases inter-tissue contrast. Conversely, in VNC and high keV VMI, the inter-tissue contrast of the images decreases. Secondly, the time interval between the CTA examination and the admission was about 7 and 8 days for AD and IMH patients, respectively, and the hematomas were not fresh, with attenuation values attenuated compared to fresh hematomas. Whereas the components of thrombi were heterogeneous, different types and sources of thrombi had different amounts of their internal components [17]. Fresh thrombi were composed of fibrin and red blood cells, and over time, they transitioned from being fibrin-dominated to collagen-dominated. Inflammatory and mesenchymal cells increased concurrently, and these changes are associated with thrombus stiffness [18] and may also impact the thrombus density or CT value. Further research is needed in this area. The study suggested that irregularly shaped, tough thrombi may have gaps between the thrombus and the vessel wall, into which the contrast agent can penetrate through the porous structure [19]. There may be an insignificant difference in attenuation values between the two. However, the hematoma’s normalized iodine concentration, normalized iodine non-water concentration, and the slope of the energy spectrum curve were higher than those of the thrombus. This difference could aid in the differential diagnosis between the two.

In PI, there was no difference between the CT values of thrombus and non-calcified plaques, whereas in VNC and VMI_100keV_, the CT values of thrombus were significantly higher than those of non-calcified plaques. This was mainly due to the different components of the two. Various materials have different attenuation values when X-rays pass through them. It was evidenced by the higher thrombus normalized Z-values compared to non-calcified plaques, indicating a larger atomic number difference between thrombus and non-calcified plaques. The image noise of VMI and VNC, reconstructed using the spectral algorithm, was significantly lower than that of the PI. This can impact the measurement of material attenuation values.

Our study indicated that non-calcified plaques intensified significantly more than thrombi and hematomas, which were essentially non-intensified. Atherosclerosis is a chronic inflammatory disease. Vascular inflammation is a key feature of atherosclerosis formation, which precedes the formation of atherosclerotic plaques [20]. Unlike plaques and thrombi, the enhancement degree of hematoma was negative, indicating that the mean attenuation value of hematoma in PI was lower than in VNC. In this study, 82 patients exhibited lesions with attenuation values in PI were lower than those in VNC, including 37 patients with IMH. A previous study has shown that attenuation values in the VNC were not a sufficient substitute for attenuation values in true non-contrast imaging (TNC) [21]. Compared to TNC, VNC overestimates the attenuation values of blood and fat, and underestimates the attenuation values of soft tissues [22, 23]. Furthermore, the study revealed that in VNC, the CT value of the lumen was higher than that of the wall lesion in 285 patients, suggesting incomplete removal of iodine from the lumen, i.e., there was a discrepancy between the attenuation values of VNC and TNC. This finding was in line with previous studies [22–25]. The VNC is created by subtracting the attenuation caused by iodine from the enhanced image, which was based on the decomposition of two materials: iodine and water [26, 27]. Non-watery material in soft tissues will be identified as non-zero iodine and removed. However, blood in vessels has a complex composition that includes proteins, hemoglobin, and other components, resulting in increased x-ray attenuation [16].

AAS is usually associated with elevated inflammatory markers such as D-dimer or CRP [28]. However, these elevated serological markers can be caused by the underlying disease or the acute aortic lesion itself and are nonspecific [29, 30]. Research has indicated a close relationship between the formation of AD and inflammation [31]. Additionally, approximately one-third of patients with AAS have inflammation of the vessel wall [32]. Current methods for evaluating vascular inflammation depend on systemic plasma biomarkers. Neutrophils play a crucial role in regulating the inflammatory response by secreting serine proteases, histone proteases, and reactive oxygen intermediates, which can cause damage to endothelial cells, platelet aggregation, and hypercoagulable states [33]. In patients with AD, the quantity of neutrophils in the vessel wall is higher than that in normal aortic vascular tissue [34]. The formation of AD triggers an inflammatory response that leads to an increase in neutrophil levels and a decrease in lymphocyte levels. High neutrophil-to-lymphocyte ratio can be used as an inflammatory marker to predict the onset and prognosis of AD [35, 36]. The study results indicated a significant positive correlation between the normalized Z_lesion_ and neutrophil%, neutrophil count, CRP, and D-dimer. Conversely, a significant negative correlation was observed between the normalized Z_lesion_ and lymphocyte% and lymphocyte count. Although statistically significant, these correlations were weak. This may reflect the distinction between systemic laboratory markers and localized PVAT inflammation, as well as heterogeneity in lesion staging. The weak coefficients limit the clinical significance of our findings. Future studies with histopathological or PET/CT validation are needed to establish stronger correlations.

In addition, this study found differences between the attenuation values of periaortic adipose tissue surrounding different lesions, as well as differences in the spectral CT characteristics. Studies have shown that perivascular adipose tissue (PVAT) is metabolically active and releases a variety of bioactive molecules that exert endocrine and paracrine effects on the vascular wall [37, 38]. This may play a pathogenic role in mediating localized vascular disease. Antonopoulos et al. [39] demonstrated that the human vasculature has a paracrine effect on PVAT, which has been validated in coronary arteries using CT imaging methods. Several studies have shown that the fat attenuation index (FAI) of peri-coronary adipose tissue can be used to visualize coronary inflammation [39–41] and to predict cardiovascular events [42]. In contrast, periaortic adipose tissue located in the thoracic aorta was closely associated with systemic inflammation and has been linked to vascular calcification [43, 44]. This suggests that PVAT may have localized toxic effects on the vascular system [45]. Further studies are needed to investigate the relationship between periaortic adipose tissue and vascular inflammation, as well as the impact on adverse cardiovascular events.

A previous study by Dai et al. [46] did not find a correlation between FAI of coronary arteries and serum ultrasensitive C-reactive protein levels. Nonetheless, our study found a significant correlation between Z values of adipose tissue around aortic wall lesions and laboratory markers of inflammation. The reason for this difference may be that in the former, FAI was based on the mean attenuation value of the fat volume, while in the present study, measurements of the adipose tissue surrounding the lesion were based on a two-dimensional plane. Secondly, varying tube voltage settings will lead to minor differences in FAI measurements [39]. In the former scenario, the tube voltage was automatically determined by the automatic exposure regulation system (Care KV) based on the patient’s body size, whereas the tube voltage in the present study was kept constant at 120 kVp. It is necessary to further validate whether the attenuation values and spectral parameters of adipose tissue surrounding aortic lesions can accurately reflect vessel wall inflammation and aid in differential diagnosis in larger samples.

The clinical significance of this study lies in facilitating the differential diagnosis of the diseases common leading to aortic wall thickening by quantitative CTA parameters, and in identifying optimal spectral CT parameters to predict active vascular inflammation in patients with aortic disease. It is important to note that this study has some limitations. Firstly, the diagnosis of the lesions in three groups was based on aortic CTA rather than pathologic findings. The selected thrombus was from the false lumen of AD patients and did not include the mural thrombus. Thrombus in the false lumen was clinically common and easily accessible, whereas primary mural thrombi lacked pathologic confirmation in the clinic, and the time of thrombus formation was unknown. Secondly, the non-calcified plaques in the plaque group were smaller than hematomas and thrombi. It was possible that plaques were secondary to or combined with thrombi. However, it is important to note that acute rupture of an atherosclerotic plaque is what lead to local thrombosis [47]. During non-calcified plaques ROI outlining, we observed regularity without ulceration on the luminal side of the plaque at the selected level. Thirdly, the patient did not provide a sample of the aortic wall lesion for histochemical analysis or other inflammatory imaging, such as positron emission tomography, to confirm the presence of wall inflammation. Fourth, we selected a representative level of aortic lesion involvement instead of an average of all lesions and periaortic adipose tissue of the entire aorta. This may explained the moderate efficacy of quantitative spectral CT parameters in the study. Fifth, this was a retrospective, single-center study. All diagnostic thresholds were derived and evaluated on the same dataset without independent validation. Therefore, independent external validation in a multi-center cohort is required before these thresholds can be considered for clinical application. Several limitations of this study point to specific directions for future research: First, the weak correlations observed between spectral CT parameters and laboratory inflammatory markers suggest that systemic biomarkers may not adequately capture local vessel wall inflammation. Future studies should directly compare spectral CT parameters with histopathological findings, PET/CT or other inflammatory tracers to validate the biological underpinnings of these imaging biomarkers. Second, our manual ROI selection on a single cross-section is subject to selection bias and does not capture whole-lesion heterogeneity. The development of artificial intelligence-based algorithms for automated, three-dimensional lesion segmentation and texture analysis could provide more comprehensive, reproducible, and observer-independent quantification. Third, whether these spectral CT parameters can predict disease progression, complications (e.g., rupture, dissection), or response to anti-inflammatory therapy remains unknown. Longitudinal studies with serial imaging and clinical follow-up are needed to assess their prognostic value.

## Conclusions

Quantitative parameters from spectral CT can facilitate the identification of IMH, non-calcified plaques, and thrombi within the aortic wall. Furthermore, normalized Z_lesion_ of the aortic wall and Z_periaortic fat_ showed significant but weak correlations with serological inflammatory markers, suggesting a potential role for these parameters in the non-invasive assessment of local inflammation. These findings are exploratory and require further validation. If confirmed, this approach may complement conventional CTA in the evaluation of aortic wall thickening diseases and potentially guide targeted anti-inflammatory therapy.

## Supplementary Information

Below is the link to the electronic supplementary material.

Supplementary Material 1

## Data Availability

The data presented in this study are available on reasonable request from the corresponding author.

## Abbreviations

AAS: Acute aortic syndrome
AD: Aortic dissection
AS: Aortic atherosclerosis
CRP: C-reactive protein
CT: Computed tomography
CTA: Computed tomography angiography
DECT: Dual-energy CT
DLCT: Dual-layer detector spectral CT
ID: Iodine density
IMH: Intramural hematoma
IW: Iodine non-water density
LDL: Low density lipoprotein
NID: Normalized iodine density
NIW: Normalized iodine non-water density
PI: Polyenergetic image
ROC: Receiver operating characteristic curve
ROI: Region of interest
SD: Standard deviation
SBI: Spectral based images
VMI: Virtual monoenergetic image
VNC: Virtual non-contrast image
Z: Effective atomic number
