# Supplementary material for: Quantitative assessment of aortic wall thickening disease by dual energy computed tomography angiography
Source: BMC Med Imaging. 2026 Jul 24;26:545. doi: 10.1186/s12880-026-02605-8 (PMC13700438; doi:10.1186/s12880-026-02605-8)
Supplement: Supplementary file 1 [file 12880_2026_2605_MOESM1_ESM.docx]

**Supplementary**

Figure 1. Schematic diagrams of ROI delineation for the three patient groups. (a–d) A patient with AD thrombosis; (e–h) a patient with intramural hematoma (IMH); (i–l) a patient with atherosclerosis. The image types are as follows: (a, e, i) polyenergetic images (PI); (b, f, j) virtual non-contrast (VNC) images; (c, g, k) 40 keV virtual monoenergetic images (VMI); (d, h, l) 100 keV VMIs.
